# Multiple vulvar epidermal inclusion cysts: a case series and discussion of management

**DOI:** 10.1097/JW9.0000000000000166

**Published:** 2024-07-16

**Authors:** Winnie Fan, Melissa A. Levoska, Jessica G. Labadie

**Affiliations:** a Department of Dermatology, University of California, San Francisco, School of Medicine, San Francisco, California; b Department of Dermatology, Icahn School of Medicine at Mount Sinai, New York, New York

**Keywords:** benign vulvar neoplasm, epidermal inclusion cyst, vulvar dermatology, vulva

**What is known about this subject in regard to women and their families?**

- Vulvar lesions, even when benign, can elicit anxiety in women due to the sensitivity of the region.
- Vulvar epidermal inclusion cysts are benign cysts thought to be caused by skin trauma and blocked follicles.
- When these vulvar cysts become inflamed or infected, they can cause discomfort during sexual activities, sports, or other activities.

**What is new from this article as messages for women and their families?**

- Multiple vulvar epidermal inclusion cysts were linked to triggers (eg, trauma or surgery) and generally reported as asymptomatic.
- In our case series, the lesions present idiopathically and caused vulvar pruritus, leading to lichen simplex chronicus and affecting the cosmetic appearance of the vulva.
- This case series explores clinical presentation and symptoms related to multiple vulvar epidermal inclusion cysts and provides information on management options.

Vulvar epidermal inclusion cysts (EICs) can be difficult to manage due to the anatomic variation of external genitalia and patients’ functional and cosmetic concerns.^1^ Herein, we discuss the presentations and management of 2 patients with multiple vulvar EICs.

Case 1 is a 28-year-old African American woman who was referred by her gynecologist for severe, chronic vulvar itching. On exam, she had hyperpigmented, lichenified plaques on bilateral labia majora and faint scaling of the inguinal creases. A KOH smear was negative. She was started on clobetasol ointment for lichen simplex chronicus (LSC). Seven months later, the patient returned with symptom improvement after as-needed usage of clobetasol ointment, though with new complaints of numerous (>20) 2 to 5 mm visible yellow cysts on the glabrous skin of the labia majora. A punch biopsy was consistent with EIC. Over the course of a year, she underwent a total of 35 punch excisions, with 4 to 6 excisions performed per visit using a 3 to 5 mm punch biopsy tool, depending on cyst size. The excisions were spaced 1 to 2 weeks apart. The surgical wounds were reapproximated with 4-0 vicryl (removed at 1 week) and wound care with twice daily petroleum jelly and gauze. At her follow-up appointments, she reported that her pruritus and self-esteem improved (Fig. 1A–C). Case 2 is a postmenopausal African American 59-year-old woman, who presented with chronic vulvar pruritus, discomfort, and “bumps.” On exam, she had lichenification and hyperpigmentation of the bilateral labia majora, consistent with LSC, along with numerous (>15) mobile dermal cysts ranging 2 to 5 mm on the glabrous skin of the labia majora. Biopsy showed EIC and the patient opted for punch excision of the left lower labial cysts (Fig. 2A and B). Over the course of 3 months, she elected to remove a total of 12 cysts (using the same approach as in case 1) and reported improvement in both symptoms and self-esteem at follow-ups.

**Fig. 1.**
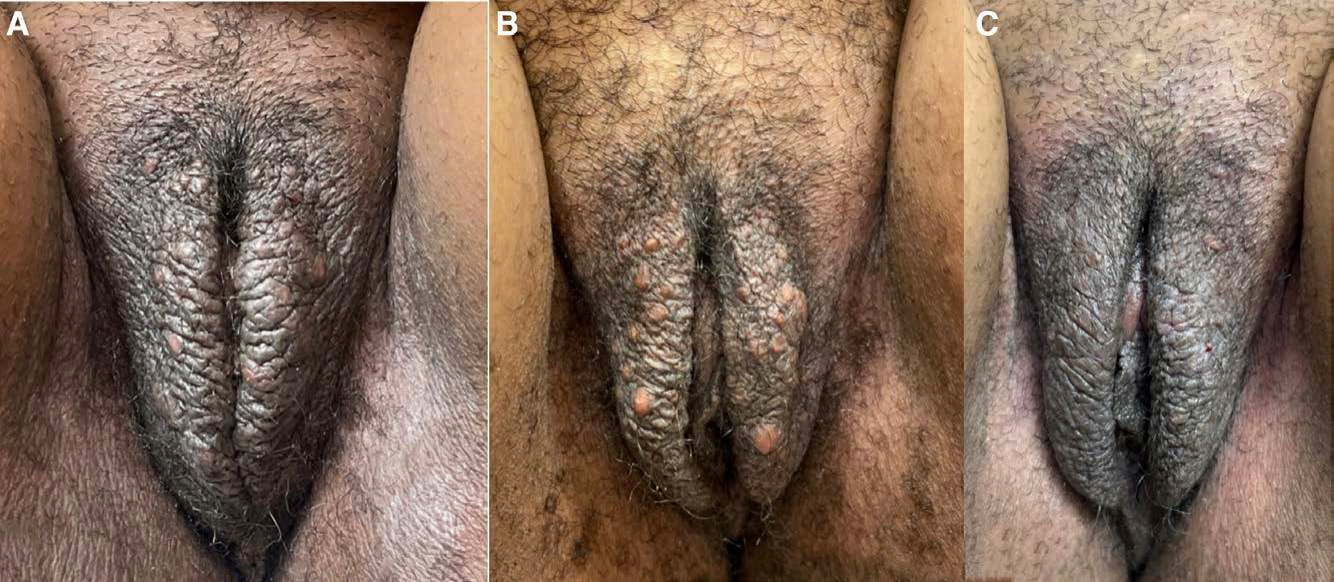
Case 1 baseline photo (A). There are hyperpigmented lichenified plaques on the bilateral labia majora. After treatment, several 2–5 mm mobile dermal cysts were unveiled (B). After punch excision, the patient noted improvement in symptoms and improved self-esteem (C).

**Fig. 2.**
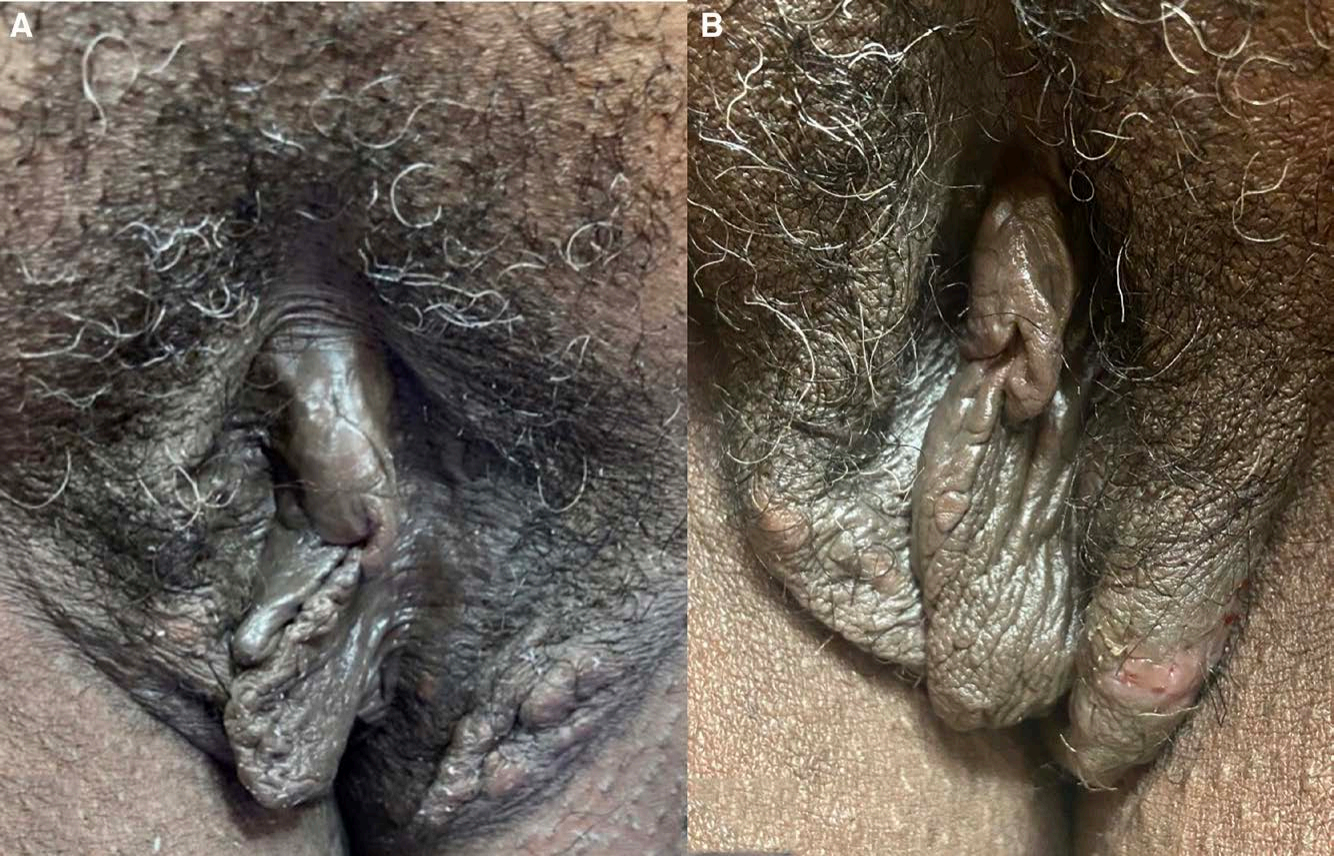
Case 2 before (A) and after (B) punch excision of cysts. The patient desired to have only the left lower labia area treated. She noted improved hyperpigmentation and lichenification, as well as improved self-esteem.

This case series reports uncommon presentations of multiple vulvar EICs and highlights their association with pruritus, discomfort, and subjective aesthetic concerns. Both were successfully treated with punch excisions.

Vulvar EICs may be underreported by patients due to the sensitivity of the region. Moreover, the labia majora frequently goes unexamined during total body skin examinations, leading to delayed diagnoses.^2^ Surgical interventions such as genital mutilation or episiotomy have been suggested as causes of vulvar EICs.^3^ Neither of our patients had a history of trauma or procedure. Notably, in all previous reports, vulvar EICs were asymptomatic or caused discomfort due to growth or swelling. Pruritus, as in our patients, was not noted. Our case series also emphasizes the importance of reexamining the patient posttreatment for vulvar LSC to rule out the primary underlying cause, which for our one patient was multiple EICs.

EICs, though benign, rarely resolve on their own. Treatments aim to preserve genital function and cosmesis while minimizing long-term complications such as recurrence or infection. Extra precaution should be taken when EIC is located close to the clitoral location or the urethral meatuses. Incision and drainage provide good cosmetic results, though with a high reoccurrence rate. Literature on the use of electrocautery and ablative laser surgery is limited and inconclusive.^4^ Both laser and electrocautery, when used at depths necessary for complete cyst wall removal, may cause significant tissue damage and extended healing time when compared to punch excision. Our study suggests that punch excision is a safe, simple, and effective approach for treating multiple vulvar EICs.

Overall, punch excision appears to be a promising approach for treating multiple vulvar EICs, especially considering its accessibility and positive cosmetic outcomes.

## Conflicts of interest

None.

## Funding

None.

## Study approval

N/A

## Author contributions

WF: Participated in data analysis and writing of the paper. MAL: Participated in research design and writing of the paper. JGL: Participated in research design, writing of the paper, and performance of the research.

## Patient consent

Informed, written consent was received from all patients for whom photographs are present in the manuscript.
